# Building fault detection data to aid diagnostic algorithm creation and performance testing

**DOI:** 10.1038/s41597-020-0398-6

**Published:** 2020-02-24

**Authors:** Jessica Granderson, Guanjing Lin, Ari Harding, Piljae Im, Yan Chen

**Affiliations:** 1 Lawrence Berkeley National Laboratory, Berkeley, USA; 2 Oak Ridge National Laboratory, Oak Ridge, USA; 3 Pacific Northwest National Laboratory, Richland, USA

**Keywords:** Energy science and technology, Mechanical engineering

## Abstract

It is estimated that approximately 4–5% of national energy consumption can be saved through corrections to existing commercial building controls infrastructure and resulting improvements to efficiency. Correspondingly, automated fault detection and diagnostics (FDD) algorithms are designed to identify the presence of operational faults and their root causes. A diversity of techniques is used for FDD spanning physical models, black box, and rule-based approaches. A persistent challenge has been the lack of common datasets and test methods to benchmark their performance accuracy. This article presents a first of its kind public dataset with ground-truth data on the presence and absence of building faults. This dataset spans a range of seasons and operational conditions and encompasses multiple building system types. It contains information on fault severity, as well as data points reflective of the measurements in building control systems that FDD algorithms typically have access to. The data were created using simulation models as well as experimental test facilities, and will be expanded over time.

## Abstract (summary)

**Table:** 

Measurement(s)	building • heating • air conditioning • fault • temperature of air
Technology Type(s)	Sensor • computational modeling technique
Factor Type(s)	mode • day
Sample Characteristic - Environment	building

Machine-accessible metadata file describing the reported data: 10.6084/m9.figshare.11743074

## Background & Summary

Buildings use 40% of primary energy globally, and account for 33% of direct and indirect carbon emissions from fuel combustion^1^. In US commercial buildings, an average 29% of energy use can be reduced through more efficiency operations and improved controls^2^. Algorithms developed to perform automated fault detection and diagnostics (FDD) use building operational data to identify the presence of faults and (in some cases) isolate their root causes.

As buildings become more data rich, and as data science comes to buildings, FDD is of increasing relevance to the building community. Outside of the research community, building owners and operators at the leading edge of technology adoption are using FDD to enable median whole-building portfolio savings of 7%^3^. Modern commercial and research-grade FDD technologies often integrate with building automation systems (BAS) to obtain operational controls data for their algorithms, or are implemented as retrofit add-ons to existing equipment. Extensive libraries of detection logic are continuously run against the data, and results are surfaced through a graphical user interface for resolution by operations and maintenance staff^4^.

A diversity of techniques is used for FDD in buildings, spanning physical models, black box, and rule-based approaches and researchers continuously strive to develop new and better algorithms, with hundreds of methods published in the literature^5^. A persistent challenge has been the lack of common datasets and test methods to support the development of, and to benchmark the performance accuracy of FDD methods against one another. Prior work has made progress toward common test methods^6,7^, however test datasets remain a gap.

Overall, there are limited examples of publicly available *operational* datasets for building energy efficiency applications. For example, utility smart meter data, HVAC control system data, lighting system data, and submetered electricity and gas data are often obtained on a research-project specific data, and restricted by NDAs or other data sharing restrictions. There is a nascent body of shared operational datasets for buildings, including for example^8–10^.

Specific to FDD applications, it is extremely rare to find datasets that have verified ground truth information on the presence and absence of faults. The majority of buildings have not yet implemented FDD, and often faults go undetected. Where FDD tools *have* been implemented, the historians do not indicate whether detected faults were verified, and false positives and negatives may confound interpretation of the historic records. While BAS are common in larger buildings, and may contain time series trend logs of operational data in their historians, these data are not labeled to indicate whether it represents faulted, un-faulted, or simply atypical/anomalous system operational states.

The dataset described in this article contains operational building heating ventilation and air-conditioning (HVAC) data, paired with validated ground-truth information as to the presence and absence of faults. This dataset spans a range of seasons and operational conditions and encompasses multiple building system types, fault types, and fault severity, or intensity levels. The systems of focus include air-handling units (AHUs) and rooftop units (RTUs). The included data points reflect measurements that are typically logged in building control systems. The dataset comprises both simulated (i.e., modeled) data, and experimental (i.e., physical) data from test facilities. The data were provided by multiple contributors, and synthesized into a single repository with a common format and documentation.

The test dataset can be used by FDD developers, FDD users, and research funders to:

- Compare and contrast performance accuracy across FDD algorithms
- Identify performance gaps to focus future development efforts and resource investment
- Develop an understanding of how FDD technology overall is improving over time

A preliminary illustration of use of the dataset to compare and contrast FDD algorithm performance accuracy and identify performance gaps is documented in^11^. This initial dataset will be expanded over time to cover a larger range of operational conditions, fault types, and seasons. It will also be evolved to include a larger set of HVAC systems, specifically, chiller and boiler plants, dual-duct AHUs, terminal variable air volume (VAV) boxes, and terminal fan coil units.

## Methods

The dataset^12^ comprises 5 AHU and RTU HVAC system types, created either through simulation, or in physical experimental facilities by multiple contributors. In the following sections we describe: the tools and facilities used to create the data, system configurations and control sequences, fault profiles (type, intensities and durations), and methods of fault imposition.

### Facilities and simulation tools

The simulated datasets were created using HVACSIM+ and an EnergyPlus-Modelica co-simulation. HVACSIM+ was developed by the US National Institute for Standards and Technology^13^, the Modelica Buildings Library^14^ is developed by the Lawrence Berkeley National Laboratory, and EnergyPlus^15^ is developed by several contributors through funding from the US Department of Energy. Described with respect to other modeling tools in^16^, HVACSIM+, Modelica, and EnergyPlus are non-proprietary tools to model the behavior of building HVAC systems using physics-based approaches.

The experimental datasets were created using three experimental research facilities. Located at the Lawrence Berkeley National Laboratory in Berkeley, California, FLEXLAB^®^ ^17^ is designed to evaluate the efficacy of major building systems, individually or as an integrated whole, under real-world conditions. FLEXLAB testbeds can monitor and assess heating, ventilation, air conditioning, lighting, windows, building envelope, control systems and plug loads in any combination. Each building in the facility features to identical paired test cell. The facility is operated through a National Instruments control and data acquisition platform. The test cell used to create the dataset described in this paper comprised a 20- by 25-foot (6.1- by 7.6-meter) zone served by a 10-ton (35.2-ton) direct expansion chiller shared with the adjacent cell. The test cell contains a dedicated air-handling unit with water-sourced heating and cooling coils and a direct-drive variable frequency drive (VFD) controlled fan. Heating water is provided by a natural gas boiler. The test cell features a south-facing windowed wall, insulated concrete slab, and otherwise near-adiabatic walls and roof.

Two experimental data sets were taken in FLEXLAB, one in single-zone constant air volume (CAV) mode and one based on ASHRAE Guideline 36^18^ single-zone variable air volume (VAV) mode. Both were implemented in National Instruments’ Test Stand programming environment. The CAV mode included a modulating, staged economizer for cooling mode.

Located at the Oak Ridge National Laboratory in Oak Ridge Tennessee, the Flexible Research Platform (FRP)^19^ is a two-story building with 10 conditioned zones and 2 unconditioned zones (i.e., stair case). The building is 13.4 × 13.4 m with a 40.6 cm thick exterior wall. The 10 conditioned zones comprise eight perimeters and two cores areas. The multi-zone HVAC system used for the data described in this paper incorporates a 44 kW RTU and a natural gas furnace. Each room in the FRP has a variable-air-volume (VAV) box with electric resistance reheat. The central fan in the air-handling unit draws return air from each room. To create the data, the original intake for the fresh air in the RTU was blocked (i.e., no ventilation air) and an exhaust fan (with a known air flow rate) was left un-operated. The facility uses a dedicated Johnson Controls Metasys building automation system, through which the room set point temperatures, schedules, and other controls were implemented.

Located at the Iowa Energy Center in Ames City, Iowa, the Energy Resource Station facility was built to compare different energy efficiency measures and monitor their energy consumption and performance. The test system was controlled by a commercially available building automation system. The fault tests were conducted on an AHU serving three perimeters, and one interior zone. The system was configured to provide variable air volume space conditioning. The chilled water is provided from an air-cooled chiller and the heating water is provided by a natural gas-fired boiler. More detailed information about this facility is provided in^20^.

### System configurations and control sequences

#### Single-zone constant air volume (CAV) and variable air volume (VAV) AHU

**Fig. 1:**
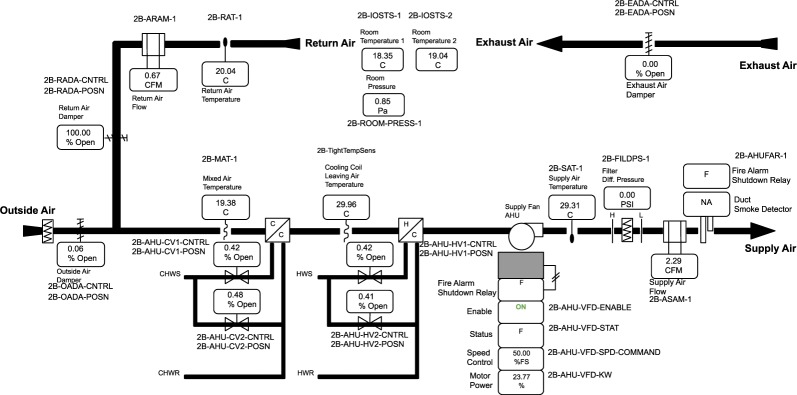
Schematic diagram of the single-zone AHU.

Figure 1 contains the schematic representation of the single-zone AHU.

Control sequence for the CAV configuration: The AHU is scheduled for automatic operation on a time of day basis for occupied and unoccupied mode. There is no dehumidification control.

Occupied mode (Monday–Sunday 6 am–6 pm)

- Fan status: The supply fan continues to run.
- Supply air temperature control: the cooling coil valve and heating coil valve shall modulate to maintain a SAT setpoint. The SAT setpoint is reset within T_min (10 °C) and T_max (30 °C) based on zone demand.
- Supply air fan speed control: The supply fan speed is fixed at 50%.
- Minimum outdoor air control: When the unit is not in economizer mode, the OA damper shall be fixed at a minimum OA damper position (15% opening).
- Economizer mode: The AHU shall enter economizer mode when outdoor air temperature is 2 °C lower than the return air temperature. The OA damper will gradually open to 100%, then RA damper will gradually close to 0% and EA damper will gradually open to 100%.
- Space temperature control: The zone heating and cooling setpoint are 21.7 °C and 23.3 °C during the occupied time period.

Unoccupied mode

- The supply fan is on. The OA and EA damper close and the RA damper fully open.
- Unoccupied heating: zone air temperature heating setpoint is 18.3 °C.
- Unoccupied cooling: zone air temperature cooling setpoint is 26.7 °C.

Control sequence for the VAV configuration: This was an advanced sequence modified from, and based upon the ASHRAE Guideline 36. The AHU was scheduled for automatic operation on a time of day basis for occupied and unoccupied mode. There was no dehumidification control.

Occupied mode (Monday–Sunday 6 am–6 pm)

- Fan status: The supply fan continues to run.
- Supply air temperature control: In cooling mode, the heating coil valve is closed and the cooling coil valve shall modulate to maintain a SAT setpoint. The SAT cooling setpoint is reset within T_min (12.8 °C) and T_max (22.5 °C) based on zone demand; In heating mode, the cooling coil valve is closed and the heating coil valve shall modulate to maintain a supply air temperature (SAT) setpoint. The SAT heating setpoint is reset within T_min (22.5 °C) and T_max (30 °C) based on zone demand. In economizer mode, the OA damper shall modulate to maintain the SAT heating setpoint.
- Supply air fan speed control: The supply fan speed is reset between minimum (10%) and maximum speed (50% in cooling mode, 30% in heating mode) based on zone demand. The minimum speed is determined to meet the ventilation with the OA damper completely open. The maximum speed is determined to provide design heating/cooling airflow for heating/cooling mode.
- Minimum outdoor air control: When the unit is not in economizer mode, the OA damper shall be fixed at a minimum OA damper position which is reset based on supply fan speed between minimum (10%) and maximum (15%). Return air damper is fully open and exhaust air damper is fully closed.
- Economizer mode: The AHU shall enter economizer mode when outdoor air temperature is 2 °C lower than the return air temperature. The OA damper will open to 100%, while RA damper will gradually close to 0% and EA damper will gradually open to 100%.
- Space temperature control: The zone heating and cooling setpoint are 21.7 °C and 23.3 °C during the occupied time period.

Unoccupied mode

- The supply fan run at minimum speed (10%). The system operates in the same way as in occupied mode to when the space temperature beyond the unoccupied heating/cooling setpoint, and disabled when the setpoint +/−2 °C is achieved.
- Unoccupied heating: zone air temperature heating setpoint is 18.3 °C.
- Unoccupied cooling: zone air temperature cooling setpoint is 26.7 °C.

#### Multi-zone VAV AHU #1

**Fig. 2:**
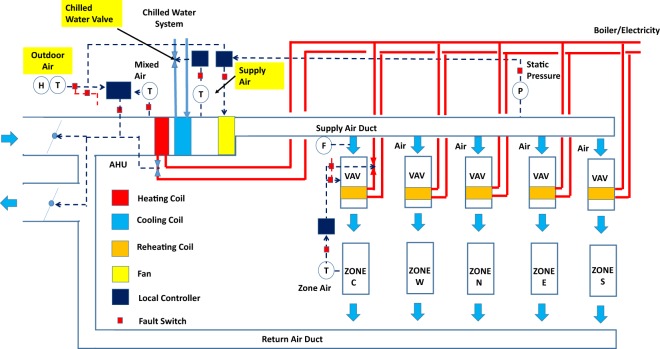
Schematic diagram of multi-zone VAV AHU #1.

Figure 2 contains the schematic representation of the multi-zone VAV AHU #1.

Control sequence: The control sequence was modified from, and based on ASHRAE 90.1–1989^21^ and 1999^22^. The AHU was scheduled for automatic operation on a time of day basis for occupied and unoccupied mode. There was no dehumidification control.

Occupied mode (Monday–Saturday 6 am–8 pm)

- Fan status: The supply fan and return fan starts or continue to run.
- Supply air temperature control: The cooling coil valve shall modulate to maintain a fixed 12.7 °C supply air temperature setpoint.
- Static pressure control: The supply fan VFD shall modulate to maintain a fixed 250 pa static pressure set point. Return fan VFD is controlled as the same as supply fan.
- Minimum outdoor air control: When the unit is not in economizer mode, the OA damper shall be fixed at a minimum OA damper position (14% opening)
- Economizer mode: The AHU shall enter economizer mode when outdoor air temperature is below 15.5 °C. The OA damper will modulate in sequence with return air damper to maintain the supply air temperature setpoint. The cooling coil valve will be closed. Once the OA damper is greater than 100% open. The cooling coil valve shall be enable to maintain supply air temperature setpoint
- VAV box reheating coil valve and airflow control: In cooling mode, when the zone cooling setpoint is met, VAV airflow is 30% of max flow rate; when the zone temperature is −1.7 °C higher than the setpoint, the damper is 100% open or max airflow rate, when zone temperature is between the setpoint and setpoint +1.7 °C, the damper modulate so that the airflow rate is between 30% and 100% of max flow rate. In heating mode, VAV airflow is 30% of max flow rate, when the zone temperature is −1.7 °C lower than the setpoint, the heating coil valve is fully open; when the zone temperature is at zone temperature heating setpoint, the heating coil valve is 0% open. When zone temperature is between the heating setpoint and setpoint −1.7 °C, the heating coil modulate between 0% to 100%. When the zone temperature is between zone cooling setpoint and heating setpoint, VAV airflow is 30% of max flow rate.
- Space temperature control: The zone heating and cooling setpoint are 21.1 °C and 23.8 °C during the occupied time period.

Unoccupied mode

- Fan status: The supply fan is off. The cooling coil valve closes and the OA damper close. The return fan is controlled as the same as supply fan. System cycling ON and OFF to maintain the unoccupied heating and cooling setpoint.
- Unoccupied heating: zone air temperature heating setpoint is 15.5 °C.
- Unoccupied cooling: zone air temperature cooling setpoint is 26.6 °C

#### Multi-zone VAV AHU #2

**Fig. 3:**
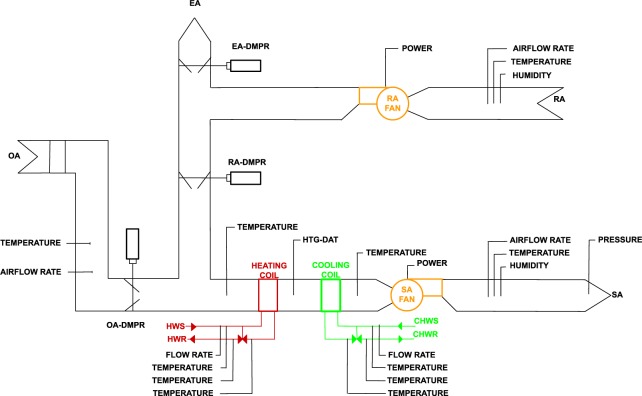
Schematic diagram of multi-zone VAV AHU #2.

Figure 3 contains the schematic representation of the Multi-zone AHU #2. An experimental dataset and a simulated data set were created based on Multi-zone AHU #2.

Control sequence: The AHU is scheduled for automatic operation on a time of day basis for occupied and unoccupied mode. There is no dehumidification control.

Occupied mode (Monday–Sunday 6 am–6 pm)

- Fan status: The supply fan and return fans start or continue to run.
- Supply air temperature control: The cooling coil valve shall modulate to maintain a fixed 12.7 °C supply air temperature setpoint. When the outdoor air damper is in the minimum position and mechanical heating is required (output from the PI control algorithm drops below 0) causing the control sequence to switch to the mechanical heating mode. During the mechanical heating mode, the valve for the AHU heating coil is modulated to maintain a fixed 18.3 °C supply air temperature setpoint.
- Static pressure control: The supply fan VFD shall modulate to maintain a fixed 9653 Pa static pressure set point. The return fan is operated with a speed tracking control sequence (80% of supply fan speed).
- Minimum outdoor air control: When the unit is not in economizer mode, the OA damper shall be fixed at a minimum OA damper position (40% opening)
- Economizer mode: The AHU shall enter economizer mode when outdoor air temperature is below 18.3 °C. The OA damper will modulate in sequence with return air damper to maintain the supply air temperature setpoint. The cooling coil valve will be closed. Once the OA damper is greater than 100% open. The cooling coil valve shall be enable to maintain supply air temperature setpoint
- VAV box reheating coil valve and airflow control: If zone temperature is less than zone heating setpoint, then a heating case exists. The VAV damper is regulated to maintain a minimum air flow rate, determined either for indoor air quality or equipment limitations. The reheating valve is regulated by a dual PI (DPI) algorithm to supply enough heated water flowing through the reheating coil to increase the entering air temperature to bring zone air temperature above heating setpoint. When zone air temperature is higher than the zone cooling setpoint, then a cooling case exists. The reheating valve position is at 0%. The VAV damper is opened to bring in more supply air to cool the zone. The air flow rate entering the zone may be varied between the minimum value and the maximum value, which is the rated maximum flow rate for the VAV unit. An air flow rate setpoint is determined by scaling the DPI output between minimum and maximum values. Another PI then regulates the damper position to maintain air flow rate setpoints. Maximum air flow rate is 1699 cubic m/hr for exterior zones and 680 cubic m/hr for interior zones. Minimum air flow rate is 340 cubic m/hr for all zones
- Space temperature control: The zone heating and cooling setpoint are 21.1 °C and 22.2 °C during the occupied time period.

Unoccupied mode

- The fans are turned off, and the dampers and valves are indexed to a fully closed position. Fully closed dampers and valves refers to 100% return air with both the heated and chilled water valves closed.

#### Rooftop unit

**Fig. 4:**
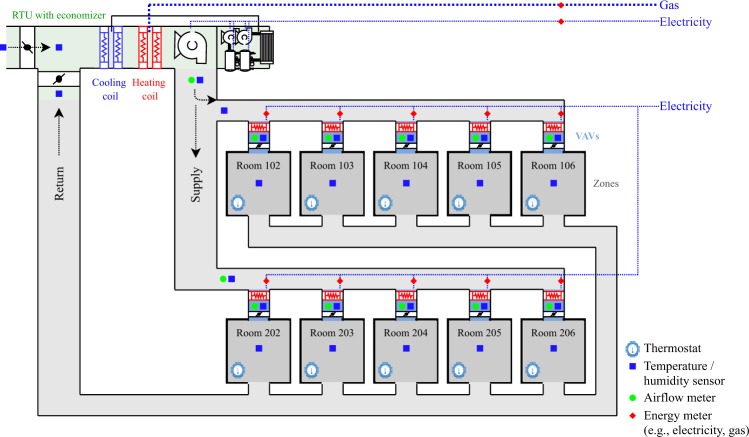
Schematic diagram of the RTU and connected 10 VAV boxes serving 10 zones.

Figure 4 contains the schematic representation of the RTU.

Control sequence for the RTU: The AHU is scheduled for automatic operation on a time of day basis for occupied and unoccupied mode.

Occupied mode (Monday–Sunday 7 am–10 pm)

- Supply air temperature control: the cooling coil valve and heating coil valve shall modulate to maintain a SAT setpoint. The SAT setpoint is 12.7 °C year-round
- Space temperature control: The zone heating and cooling setpoint are 21 °C and 24 °C during the occupied time period
- The exception was for the condenser fouling test, for which occupied mode started at 6:38am and ended at 9:38 pm

Unoccupied mode

- Unoccupied heating: zone air temperature heating setpoint is 15.5 °C
- Unoccupied cooling: zone air temperature cooling setpoint is 26.6 °C

### Fault profiles

Table 1 summarizes the faulted and unfaulted scenarios for the AHU data, including the fault type, intensity, and season in which data were acquired. Each cell contains the number of days for which data were acquired for each scenario. Table 2 contains the same information for the RTU data.

**Table 1:** Fault profiles for the AHU data; Sim = Simulated, Exp = Experimental, Sp = Spring, Sum = Summer, F = Fall, W = Winter.

Input Scenarios			MZVAV AHU-1 (Sim)				MZVAV AHU-2 (Exp)		MZVAV AHU-2 (Sim)			SZCAV AHU (Exp)	SZVAV AHU (Exp)
Fault Type		Fault Intensity	Sp	Sum	F	W	Sp	Sum	Sp	Sum	W	W	Sum
OA Damper	Stuck	Min position							1		1		1
		Fully open										1	1
		40% open							1				
		45% open								1			
		50% open								1		1	
Valve of Heating Coil	Stuck	Fully Closed										1	
		50% open										1	1
		Fully Open										1	1
	Leaking	Low						1		1		1	
		Medium						1		1			
		High						1		1		1	1
Valve of Cooling Coil	Stuck	Fully Closed							1			1	
		Fully Open							1	1		1	1
		15% Open								1			
		50% Open										1	
		65% Open								1			
	Leaking	Low										1	
		High										1	1
Outdoor Air Temperature	Bias	+1 °C	7	7	7	7							
		+2 °C	7	7	7	7							
		+4 °C	7	7	7	7							
		−1 °C	7	7	7	7							
		−2 °C	7	7	7	7							
		−4 °C	7	7	7	7							
Unfaulted			7	7	7	7	4	3	3	9	1	1	4

**Table 2:** Fault profiles for the RTU data; Sum = Summer, F = Fall, W = Winter.

Input scenarios		Season		
Fault type	Fault intensity	Sum	W	F
Condenser fouling	25% reduction in condenser coil air flow full load	1		
	50% reduction in condenser coil air flow full load	1		
HVAC setback error: delayed onset	3-hour onset delay		1	
HAV setback error: early termination	3-hour early termination		1	
Excessive infiltration	+20% infiltration		1	
	+40% infiltration		1	
Lighting Setback Error: Delayed Onset	3-hour onset delay		1	
Lighting Setback Error: Early Termination	3-hour early termination		1	
No Overnight HVAC Setback	No setback		1	
No Overnight Lighting Setback	No setback		1	
Thermostat measurement bias	Bias of +2.2 °C (Core zone 103)		1	
	Bias of −2.2 C (Core zone 103)		1	
	Bias of +2.2(Perimeter zone 205)		1	
	Bias of −2.2(Perimeter zone 205)		1	
Unfaulted			6	1

### Methods of fault imposition

Tables 3 through 8 summarize how each fault was imposed for each of the represented systems and fault scenarios.

**Table 3:** Methods of fault imposition for each of the single-zone CAV AHU faults.

Input Scenarios			Method of fault imposition
Fault type		Fault intensity	
OA damper	Stuck	Fully open (100%)	Automated override of control signal values to indicate that OA damper is stuck.
		Partially open (50%)	
	Leaking	20% of max damper flow	If control signal drops below X%, fix control output at X%. Otherwise damper controls normally. X = 2 at 20% intensity, and = 10 at 50% intensity
		50% of max damper flow	
Valve of Heating Coil	Stuck	Fully closed (0%)	Automated override of control signal values to indicate that heating coil valve is stuck.
		Fully open (100%)	
		Partially open (50%)	
	Leaking	5% of max coil valve flow	Open heating coil bypass valve to 5%/40% of the maximum heating coil valve flow.
		40% of max coil valve flow	
Valve of Cooling Coil	Stuck	Fully closed (0%)	Automated override of control signal values to indicate that cooling coil valve is stuck
		Fully open (100%)	
		Partially open (50%)	
	Leaking	5% of max coil valve flow	Open cooling coil bypass valve to 5%/50% of the maximum cooling coil valve flow.
		50% of max coil valve flow	
Unfaulted			—

**Table 4:** Methods of fault imposition for each of the single-zone VAV AHU faults.

Input Scenarios			Method of fault imposition
Fault type		Fault intensity	
OA Damper	Stuck	Minimum position	Automated override of control signal values to indicate that OA damper is stuck.
		Fully open (100%)	
Valve of Heating Coil	Stuck	Fully open (100%)	Automated override of control signal values to indicate that heating coil valve is stuck.
		Partially open (50%)	
	Leaking	40% of max coil valve flow	Open heating coil bypass valve to 40% of the maximum heating coil valve flow.
Valve of Cooling Coil	Stuck	Fully open (100%)	Automated override of control signal values to indicate that cooling coil valve is stuck
	Leaking	50% of max coil valve flow	Open cooling coil bypass valve to 50% of the maximum cooling coil valve flow.
Unfaulted			—

**Table 5:** Methods of fault imposition for each of the multi-zone VAV AHU #1 faults.

Input Scenarios		Method of fault imposition
Fault type	Fault intensity	
Outdoor air temperature sensor bias (x is the true value, x′ is the faulted value)	x′ = x + 1(°C)	Add bias to sensor output
	x′ = x + 2(°C)	
	x′ = x + 4(°C)	
	x′ = x − 1(°C)	
	x′ = x − 2(°C)	
	x′ = x − 4(°C)	
Unfaulted		—

**Table 6:** Methods of fault imposition for each of the multi-zone VAV AHU #2-1 faults.

Input Scenarios			Method of fault imposition
Fault type		Fault intensity	
Valve of Heating Coil	Leaking	Stage 1: 1.5 SLM (Standard liter per minute)	Manually open heating coil bypass valve
		Stage 2: 3.7 SLM	
		Stage 3: 7.5 SLM	
Unfaulted			—

**Table 7:** Methods of fault imposition for each of the multi-zone VAV AHU #2-2 faults.

Input Scenarios			Method of fault imposition
Fault type		Fault intensity	
OA Damper	Stuck	Fully closed	Automated override of control signal values to indicate that OA damper is stuck.
		40% open	
		45% open	
		55% open	
Valve of Heating Coil	Leaking	Stage 1: 1.5 SLM	Manually open heating coil bypass valve
		Stage 2: 3.7 SLM	
		Stage 3: 7.5 SLM	
Valve of Cooling Coil	Stuck	Fully closed	Automated override of control signal values to indicate that cooing coil valve is stuck.
		Fully open	
		Partially open 15%	
		Partially open 65%	
Unfaulted			—

**Table 8:** Methods of fault imposition for each of the rooftop unit faults.

Input Scenarios		Method of fault imposition
Fault type	Fault intensity	
Condenser Fouling	25% reduction in condenser coil air flow full load	Cover the condenser face using screen, mesh, or cloth
	50% reduction in condenser coil air flow full load	
HVAC Setback Error: Delayed Onset	3-hour onset delay	Modify the control programming
HVAC Setback Error: Early Termination	3-hour early termination	Modify the control programming
Excessive infiltration	+20% infiltration	Open windows to achieve target infiltration area
	+40% infiltration	
Lighting Setback Error: Delayed Onset	3-hour onset delay	Modify the control programming
Lighting Setback Error: Early Termination	3-hour early termination	Modify the control programming
No Overnight HVAC Setback	No setback	Modify the control programming
No Overnight Lighting Setback	No setback	Modify the control programming
Thermostat measurement bias	Bias of +2.2 °C (Core zone 103)	Adjust the temperature set point
	Bias of −2.2 °C (Core zone 103)	
	Bias of +2.2 °C (Perimeter zone 205)	
	Bias of −2.2 °C (Perimeter zone 205)	
Unfaulted		—

## Data Records

The data are stored on figshare and on OpenEI, a wiki-based platform that supports public sharing of, and access to data and analyses related to renewable energy and energy efficiency. Summarized in Table 9, the dataset^12^ comprises a collection of six comma separated value (CSV) files. Each CSV file represents a single combination of system configuration and experimental or simulated data creation approach. The data are minute-frequency time series measurements of the system operational parameters that are most commonly available to FDD algorithms in typical commercial buildings. Time stamps are in the first column of each file, and presented in the format *m/d/yy h:mm*. The final column of each file contains a binary indicator of the ground truth information on whether or not a fault is present.

**Table 9:** Files and size of each file in the full dataset, as well as system of focus and provenance.

Data file	System	Data provenance	Total file size
SZVAV	Air handling unit: single zone variable air volume	Experimental	1.2 MB
SZCAV	Air handling unit: single zone constant air volume	Experimental	1.6 MB
MZ-VAV-2-1	Air handling unit: multi-zone variable air volume	Experimental	1.8 MB
MZ-VAV-2-2	Air handling unit: multi-zone variable air volume	Simulation	2.9 MB
MZ-VAV-1	Air handling unit: multi-zone variable air volume	Simulation	22.7 MB
RTU	Rooftop unit	Experimental	9.9 MB

The set of CSV files is accompanied with a data ‘inventory’ file that describes:

key information necessary to understand the content and scope of each data set, including:

- An overview of the data set, who created it, and whether it was generated through simulation or physical experimentation
- Building and system information
  ○ Model or experimental facility description
  ○ System type and diagram
  ○ Control sequences
- Data points
- Input scenarios for faulted and fault-free conditions represented in the data

○ Fault types

○ Fault intensities

○ Method of fault imposition

○ Fault occurred time

## Technical Validation

The technical quality of the dataset can be understood through 3 primary lenses: 1) accuracy of measurement in the facilities; 2) accuracy of the simulation models; 3) accuracy of the ground truth information on the presence or absence of faults and their severity. Description and illustrations of each are provided in the following.

### Facilities measurement

The facility measurements that are included in the dataset comprise sensor data as well as data that indicate equipment status and control commands. Sensor data span temperature, relative humidity, power, pressure, and air flow. Equipment status and control commands encompass parameters such as valve and damper control commands, temperature setpoints, and operational modes.

Specifications of the relevant FRP and FLEXLAB sensors are provided in Table 10. Many more sensors are available in the facilities, however we report only those used in the dataset described in this paper).

**Table 10:** Specifications of relevant sensors from the FLEXLAB and FRP experimental facilities.

Facility	Sensor	Measurement	Accuracy
FLEXLAB	BAPI BA/10K-2(XP)-D-12′′-BB thermistor	Temperature	±0.1 °C
FRP	Campbell Sci HC2S3-L	Temperature, relative humidity (RH)	±0.1 °C and ±0.8% RH @ 23 °C
	Continental Controls WNB-3D-240P	Power	±0.5% of reading
	Omega PX409-750-A5V pressure transducer	Pressure	±0.08% best straight line maximum
	Air Monitor fan evaluator paired with Veltron DPT2500-Plus transmitter	Air flow	DPT2500, 0.25% of natural span, including hysteresis, deadband, nonlinearity, and nonrepeatability Fan evaluator ±2%

Facility sensors are periodically calibrated using a variety of standard approaches based on ISO/IEC 17025^23^, which specifies requirements for testing and calibration laboratories. To illustrate, a detailed example is provided for the water-bath calibration of the FLEXLAB AHU temperature sensors (thermistors).

The supply and return air temperature sensors are inserted in the appropriate duct, and the mixed air temperature sensor is placed in the AHU mixed air plenum, which is open to return air and outside air (Fig. 1). The calibration is conducted using a 0.01 °C accuracy reference sensor (US sensor USP3021), an Agilent/Keysight 34970 A data acquisition unit, and an Anova water bath. All Agilent measurements are conducted to ensure that the measurement of the testing sensor is consistent with the reference sensor, and they use temperature step sizes of 5 °C to bound the range of interest.

**Fig. 5:**
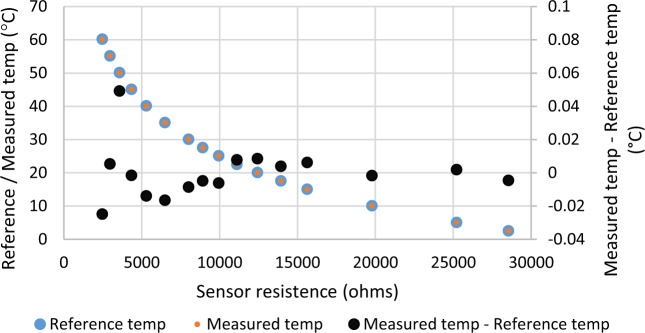
AHU supply air temperature water-bath calibration.

An example of a supply air temperature calibration result from this process is show in Fig. 5, showing calibration of experimental sensors to within 0.02 °C of the reference sensor. The normal range of interest for the temperature sensors at FLEXLAB is 5 °C to 45 °C, so the supply air temperature sensor is calibrated in a range of 5 °C to 60 °C. The blue dots and orange dots are the temperature measurements from the reference senor and the newly calibrated supply air temperature sensor respectively. The black dots which are plotted on the secondary y-axis represent the difference between the newly calibrate sensor to the reference sensor. The plot shows that across the full range of interest the measurements of the newly calibrated sensor (orange dots) match those of the reference sensor (blue dots) to within ±0.02 °C. There is one outlier (0.06 °C) at the low-resistance, high-temperature (left) side of the plot. Temperatures below about 35 °C are very closely aligned with the reference sensor, with offsets within ±0.01 °C.

To verify the data for equipment status and control commands, the results from functional tests were leveraged. The primary purpose of functional tests is to ensure that system operation is consistent with the designed control sequences, and reflective of fault-free operational behavior. A failed functional test would indicate incorrect implementation of control logic, equipment faults, or inaccurate system-reporting of status and command data.

**Fig. 6:**
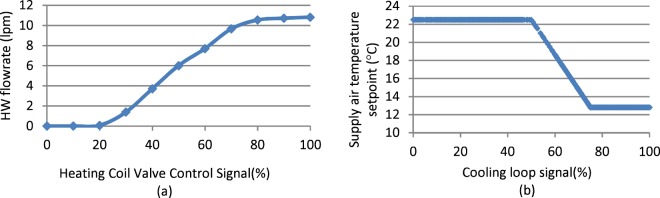
Examples of functional test results from FLEXLAB.

For example, Fig. 6(a) shows the characteristic curve for the FLEXLAB AHU heating coil valve, heating water flowrate versus heating coil valve control signal. As expected, once the valve is opened past 20%, the flowrate steadily increases until reaching its maximum value at the 80–100% open position. Similarly, Fig. 6(b) shows supply air temperature setpoint versus cooling loop control signal. It indicates that the cooling loop control signal modulates between higher and lower values to drive a supply air temperature setpoint reset in accordance with the defined control sequence.

**Fig. 7:**
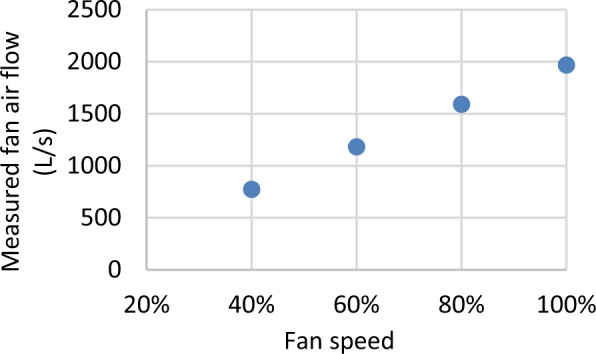
Examples of functional test results from the FRP.

Figure 7 shows a functional test from the FRP RTU, in which the control command for RTU fan speed (%) signal is observed to appropriately track with measured RTU fan airflow (L/S), as the signal is increased from approximately 40% to 100%.

### Simulation models

An EnergyPlus-Modelica model that was used to generate the simulated data for multi-zone AHU #1^24^. The US Department of Energy’s large office Commercial Reference Building Model^25^ (hereafter referred to as *Reference Building*) in EnergyPlus was used to calculate the building thermal load, as the *Reference Buildings* are taken to represent reasonably realistic building characteristics and construction practices and are widely used in the evaluation of building design and operation^26^. Since control processes are idealized in EnergyPlus, and may not capture short-term behaviors of HVAC systems, the HVAC systems in the large office *Reference Building* were re-implemented with components from the Modelica Buildings Library to model the dynamic behaviors. To retain realistic system response in the Modelica representation, the system performance curves from the *Reference Building* were transferred to the Modelica representation. Modelica component and system models have been validated using empirical validation, comparative testing and analytical verification. Most relevant to the models used in this work, comparative testing, in which results are compared with other simulators, has been used for the multi-zone airflow models^27^. In addition, analytical verification, in which results are compared with exact solutions, has been used to validate most of the individual component models in Modelica, such as for heat and mass transfer and storage, for flow resistance elements such as valves and pipes, for fan models and for radiosity transport models^28^.

The HVACSim+ model that was used to generate the simulated dataset for multi-zone VAV AHU #2 was validated with the experimental data from the physical unit that the model was created to represent. This validation is detailed in^29^. To validate the model under fault-free conditions, steady state experiments and dynamic experiments were conducted to generate the experimental data for the comparison. Energy indices (i.e. electrical energy consumed by return and supply fan, heating water energy consumed by heating coil, and chilled water energy consumed by cooling coil) were used to compare the simulated energy consumption with real energy consumption during the experiment. Operational indices (i.e. temperature, air flow, and control indexes) were used to compare the simulated operational variables with the actual measurements. To illustrate, the temperature operational indices that were used for model validation are provided in Table 11. In the Table “max diff.” indicates the maximum difference of the hourly average values between simulated values and experimental data. Maximum differences less than 20% of the typical value were taken as sufficient.

**Table 11:** Example of temperature operational indices used to validate a simulation model with experimental data.

Indices			Summer in 2007			Winter in 2008		Spring in 2008		
			8/19	8/25	9/4	2/16	2/17	5/2	5/3	5/9
Temp °C	Supply air	typical value	12.7	12.7	12.7	18.3	18.3	12.7	12.7	12.7
		max. diff.*	−0.9	−0.7	−0.5	−0.2	−0.05	−0.4	−0.8	−0.4
	Mixed air	typical value	23.8	23.3	23.3	12.7	13.3	18.3	13.3	8.3
		max. diff.	−0.4	−0.7	−0.8	−0.7	−0.2	−2.3	−1.0	−1.3

**Fig. 8:**
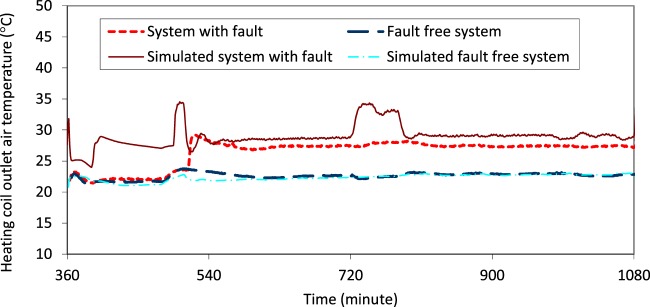
Heating coil outlet air temperature associated with the presence and absence of a leaking heating coil fault.

To validate the model for faulted conditions, the simulated operational data under a specific fault were verified to ensure that they reproduced the major fault signature obtained from the facility measurements. An example is shown in Fig. 8. The plot shows measured and simulated the heating coil outlet air temperature under faulted and un-faulted operation. The simulated temperature (brown solid line) during faulted operation is much higher than the simulated temperature during fault free operation (light blue dashed line). This offset behavior was consistent with that observed in the physical measurements, shown in red (faulted operation), and dark blue dashed (un-faulted operation).

### Fault ground truth

Ground truth assignment of the data as faulted or fault-free, was validated first through functional testing (experimental data), and engineering logic (experimental and simulated data). Described in the preceding section on *facility measurement*, functional tests ensure that system operation is consistent with the designed control sequences. Whereas a failed functional test can indicate faulted equipment (including sensors and status/command data), or incorrect implementation of control sequences, a successful functional test can verify fault-free operation. For example, Fig. 6(a) suggests the heating coil valve moves smoothly without the presence of any pre-existing stuck or leakage faults. Similarly, Fig. 6(b) indicates that the supply air temperature setpoint was reset according the defined sequence that was in place.

**Fig. 9:**
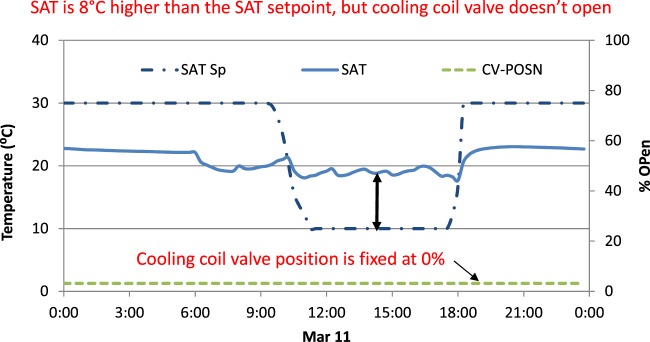
AHU operational data for an imposed stuck cooling coil valve fault.

After functional testing, the data for each fault-present or fault-free case were visually inspected to validate the presence or absence of faults (and their severity). Engineering logic and knowledge of the implemented control strategies was applied to confirm that the “fault free” scenario and “imposed fault” scenario were indeed reflected in the data trends. Figure 8 provides an example to validate the fault signature of the imposed leaking heating coil fault, in which the heating coil outlet air temperature is confirmed to increase, as compared to the fault-free system. Figure 9 illustrates another example, for a cooling coil, stuck at a severity of 100% (i.e., fully closed, or 0% open). SAT is supply air temperature; SAT Sp is supply air temperature set point; CV-POSN is cooling coil valve position. The data in the figure show that when the fault was imposed, the cooling coil valve position was overridden a fixed 0% position. The expected symptom of supply air temperature much higher than the setpoint was then observed, validating that the fault was correctly imposed.

Similar checks were conducted for each of the faults in both the experimental as well as the simulated data sets.

## Usage Notes

A complete inventory of the data was developed to support users in interpreting the content and form of the data, and the corresponding HVAC systems, controls, and faults. The data itself comprise time series that can be analyzed with whatever software tools the user elects to implement. The data are provided at 1-minute intervals, and can be resampled as needed to fit the needs of specific applications.

## Data Availability

The Modelica Buildings Library and EnergyPlus are freely available for download^30,31^. EnergyPlus runs on Windows, Mac OSX, and Linux operating systems. A Windows or Linux-based computer and Dymola solver are required to run Modelica, and Dymola can be licensed from Modelica Buildlings Library. HVACSim+ is also freely available, upon request from NIST, and has no operating system requirements. The HVACSim+ AHU model that was used in this work is available within the ASHRAE Research Project 1312 Results^29^. The data acquisition system that is implemented in FLEXLAB comprises a custom-built National Instruments platform utilizing distributed Compact RIOs (cRIOs), PC-based servers and workstations, and a Unix-based database running sMAP2.0^32^. Data are typically collected and recorded at a one-second (1 Hz) rate with averaging to one minute for database storage – suitable for most research purposes. 1 Hz data are stored in CSV files for use as needed. Control and data acquisition are implemented over the same architecture, with most control sequences running in the NI TestStand environment. A simple API also allows remote data acquisition and control using text-based messages, which allows use of other programming or scripting environments such as python or java. Experimental data are accessed from sMAP using a browser-based GUI or via text-based query. At the Flexible Research Platform, the data acquisition system utilizes Campbell Scientific data loggers. It includes measurements of the zone set point temperature and humidity, supply and return air temperature and flow rates, and energy consumption of individual components including compressor, condenser, supply fan, VAV reheating. Data are typically collected and recorded at a one-second rate with averaging thirty seconds for database storage. The data file format is CSV, with automated transfer from the data loggers to storage on an ORNL internal server, at time resolutions of 30 seconds, 1 min, 15 min, and 60 min intervals.
